# Machine learning-based prediction models for severe *Mycoplasma pneumoniae* pneumonia in Chinese children: a systematic review and meta-analysis of prediction model performance

**DOI:** 10.3389/fpubh.2026.1793116

**Published:** 2026-06-03

**Authors:** Juan Cao, Jiao Nie, Danxia Wu, Huiqin Liu

**Affiliations:** 1 College of Medicine and Health Sciences, China Three Gorges University, Yichang, Hubei, China; 2 Department of Nursing, Jiangxi Provincial Children’s Hospital, Nanchang, Jiangxi, China; 3 Department of Respiratory Medicine, Jiangxi Provincial Children’s Hospital, Nanchang, Jiangxi, China; 4 The First College of Clinical Medicine, China Three Gorges University, Yichang Central People’s Hospital, Yichang, China

**Keywords:** machine learning, meta-analysis, pediatrics, prediction model, severe *Mycoplasma pneumoniae* pneumonia

## Abstract

**Objectives:**

This study aimed to systematically evaluate the predictive performance and methodological characteristics of machine learning–based models for predicting progression to severe *Mycoplasma pneumoniae* pneumonia (SMPP) in pediatric patients.

**Methods:**

A comprehensive literature search was conducted in PubMed, EMBASE, Web of Science, Cochrane Library, CNKI, and Wanfang databases from inception to November 2025 to identify studies developing or validating prediction models for SMPP in children. Data on study characteristics, modeling algorithms, predictors, and performance metrics were extracted. A narrative synthesis was performed to summarize model characteristics, predictors, and modeling approaches, while model discrimination was quantitatively synthesized using pooled area under the receiver operating characteristic curve (AUC). Subgroup analyses were conducted according to modeling algorithms. Methodological quality and risk of bias were assessed using the PROBAST tool.

**Results:**

A total of 13 studies were included. The reported prevalence of hypoglycemia ranged from 17 to 33%. The AUC for predictive models ranged from 0.81 to 0.90. Subgroup analyses showed that machine learning-based models such as XGBoost and random forest generally reported higher AUC values compared with other modeling approaches. Commonly reported predictors included age, insulin use, body mass index, HbA1c, creatinine, and history of hypoglycemia.

**Conclusion:**

Research on risk prediction models for SMPP in children is still at a developmental stage. Although current models demonstrate high discriminatory performance, methodological limitations and limited clinical translation remain. Future studies should focus on developing robust, interpretable machine learning models and facilitating their integration into pediatric clinical practice.

**Systematic review registration:**

https://www.crd.york.ac.uk/PROSPERO/ CRD42020190338.

## 1 Introduction

Community-acquired pneumonia (CAP) remains one of the most common infectious diseases associated with high morbidity and mortality in children worldwide, and it continues to be a leading cause of death among children under 5 years of age (1, 2). Although *Streptococcus pneumoniae* has historically been the predominant pathogen in pediatric CAP, its infection rate has declined due to widespread antibiotic use, while atypical pathogens, particularly *Mycoplasma pneumoniae* (MP), have steadily increased (3, 4). Studies indicate that *M. pneumoniae* pneumonia (MPP) ranks as the third most common cause of CAP (5), presenting with diverse clinical manifestations. Accumulating evidence suggests that a significant proportion of children with MPP may progress to severe *M. pneumoniae* pneumonia (SMPP), which is associated with rapid disease progression, severe pulmonary involvement, and a high risk of multisystem complications (6, 7). Nevertheless, early identification of SMPP remains a major clinical challenge. This difficulty is largely attributable to the high heterogeneity in clinical presentation, the inconsistency between clinical signs and radiological findings, and the increasing proportion of cases in which extrapulmonary complications as the initial presentation (8). These factors significantly complicate early risk stratification of SMPP in pediatric practice, potentially leading to delayed initiation of optimal treatment and adversely affecting clinical outcomes.

With the availability of large-scale clinical data, machine learning (ML) techniques have been applied to risk prediction models for SMPP. ML-based models are capable of integrating clinical characteristics, laboratory parameters, and imaging features, enabling the modeling of complex, high-dimensional data without reliance on the restrictive assumptions of traditional statistical methods (9). Consequently, these approaches may provide valuable support for disease diagnosis, prognostic assessment, and clinical decision-making. Nevertheless, despite their promising applications in medicine, the uncertainty of ML predictions, limited model interpretability, and insufficient clinical trust have hindered the widespread adoption of artificial intelligence (AI) in routine clinical practice. Meanwhile, a growing number of ML-based prediction models for SMPP have been developed in Chinese pediatric populations; however, substantial heterogeneity exists across studies in terms of algorithms, predictor selection, sample size, outcome definitions, and validation strategies. Model performance has been reported inconsistently, with limited evaluation of calibration and scarce external validation, thereby limiting comparability across models and their potential clinical utility.

In the evaluation of ML-based prediction models, sensitivity, specificity, and the area under the receiver operating characteristic curve (AUC) are the most commonly reported and clinically interpretable performance metrics (10). Among these, AUC is widely used as a threshold-independent measure reflecting a model’s overall discriminative ability and is therefore considered the most appropriate metric for comparing predictive performance in ML models (11). However, no meta-analysis to date has systematically synthesized AUC estimates from ML-based models predicting outcomes related to SMPP, leaving the overall predictive performance and potential clinical utility of existing models unclear. Accordingly, we conducted a systematic review and meta-analysis to summarize the performance of multivariable diagnostic and prognostic prediction models for SMPP. Given the lack of consensus on a single “gold standard” definition for SMPP, all eligible diagnostic criteria were considered. The primary objective was to compare the predictive performance of competing ML-based models, while secondary objectives included identifying risk factors for SMPP and assessing modeling methods.

## 2 Material

### 2.1 Protocol and registration

We conducted a meta-analysis (PROSPERO; CRD42020190338) according to the Preferred Reporting Items for Systematic Review and Meta-Analysis of Diagnostic Test Accuracy studies (PRISMA-DTA) (12).

### 2.2 Literature search

A comprehensive search of English and Chinese literature was conducted using the databases PubMed, EMBASE, Web of Science, the Cochrane Library, CINAHL, China National Knowledge Infrastructure (CNKI), and the Wanfang Database. In addition, the reference lists of all included studies were manually screened to identify any further eligible articles. The following Medical Subject Headings (MeSH) terms and keywords were used: “*M. pneumoniae* pneumonia,” “severe *M. pneumoniae* pneumonia,” “machine learning,” “artificial intelligence,” “prediction model” and “risk prediction.” Boolean operators (“OR” and “AND”) were applied to combine search terms across different concept groups. The search period covered studies from database inception to November 2025, and only full-text original research articles were considered. All retrieved records were imported into NoteExpress software for literature management and duplicate removal. The detailed search strategy is presented in Supplementary Table 1.

### 2.3 Eligibility criteria

We applied the Population, Index prediction model, Comparator, Outcome, Timing, and Setting (PICOTS) framework (13) to define eligibility criteria: P (children with MPP), I (Machine Learning-based prediction models), C (not applicable), O (SMPP), T (early prognostic prediction during the disease course), and S (pediatric clinical settings).

The inclusion criteria were as follows: (1) children diagnosed with MPP; (2) studies focusing on the development and/or validation of prediction models for SMPP; (3) studies reporting at least one quantitative model performance metric, such as the AUC, sensitivity, or specificity; (4) study types including cohort studies, case–control studies, or other observational designs; and (5) articles written in English or Chinese. The exclusion criteria were as follows: (1) studies that only analyzed risk factors without establishing a risk prediction model; (2) studies with incomplete data or where the original texts are inaccessible; and (3) reviews, systematic reviews, meta-analyses, editorials, letters, case reports, and conference abstracts.

### 2.4 Study selection

The study selection process was conducted by two independent reviewers, who initially screened titles and abstracts to identify potentially eligible studies and remove duplicate records. Full-text articles were then independently assessed for inclusion according to the predefined inclusion and exclusion criteria, with reasons for exclusion documented. Any disagreements were resolved through discussion, and if consensus could not be reached, a third reviewer was consulted to make the final decision.

### 2.5 Data extraction

**Figure 1:**
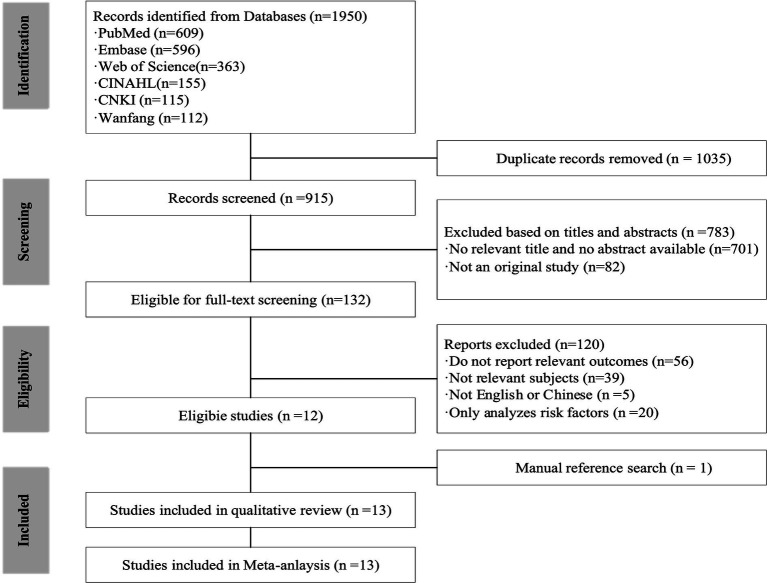
The article selection process.

Two authors (Cao and Nie) independently extracted data from all eligible studies using a standardized form based on the CHARMS checklist (14) (Checklist for Critical Appraisal and Data Extraction for Systematic Reviews of Prediction Modelling Studies). Extracted information included author names, year of publication, study design, location of research, dataset, Modeling Methods, Validation method, Model performance, model performance, and Predictors. The extracted datasets were cross-checked against the original articles, and any discrepancies were resolved through discussion; if consensus could not be reached, a third researcher was consulted for adjudication. The overall study selection process is presented in the PRISMA flow diagram (Figure 1).

### 2.6 Quality appraisal

The Prediction Model Risk of Bias Assessment Tool (PROBAST) was used to evaluate the risk of bias and applicability of the included studies. PROBAST was developed in 2019 by Wolff et al. (15) as a specialized tool for assessing studies that develop or validate prediction models. Risk of bias was assessed across four domains (participants, predictors, outcome, and analysis) using signaling questions rated as “yes/probably yes,” “no/probably no,” or “no information,” resulting in judgments of low, high, or unclear risk. A domain was rated as having a low risk of bias only if all signaling questions were judged as low risk; otherwise, the domain was considered to have a high or unclear risk of bias. Applicability was assessed across three domains, including participants, predictors, and outcome, using criteria consistent with those applied in the risk of bias assessment (16). Two reviewers independently assessed the risk of bias and applicability of the included studies using PROBAST, with disagreements resolved by a third reviewer.

### 2.7 Data synthesis

In this study, STATA 18.0 software was used to conduct the meta-analysis. Heterogeneity among the included studies was assessed using the Higgins *I*^2^ statistic, with *I*^2^ ≤ 25% indicating low heterogeneity, 25% < *I*^2^ ≤ 50% indicating moderate heterogeneity, and *I*^2^ > 50% indicating high heterogeneity. When *p* > 0.05 and *I*^2^ ≤ 50%, heterogeneity was considered acceptable and a fixed-effect model was applied; otherwise, a random-effects model was used to pool the effect sizes. Given the inherent heterogeneity of prediction modeling studies, a random-effects meta-analysis was performed (17). The AUC was used as the primary measure of predictive performance, and pooled estimates were reported with corresponding 95% confidence intervals. An AUC between 0.7 and 0.9 was considered to indicate moderate predictive accuracy, while an AUC greater than 0.9 indicated high predictive accuracy. A *p-*value < 0.05 was regarded as statistically significant. Publication bias was evaluated using Egger’s test, and a funnel plot was generated to visually assess potential publication bias (18).

## 3 Results

### 3.1 Study selection

In total, 1,950 records were identified through electronic database searches (PubMed:609, Embase:596, Web of Science:363, CINAHL:155, CNKI:115, Wanfang:112), of which 1,035 were duplicates. After screening titles and abstracts and removing duplicate records, 915 articles were assessed for eligibility. Of these, 783 studies were excluded for not meeting the inclusion criteria. Subsequently, 132 full-text articles were reviewed, and 120 were excluded due to not reporting relevant outcomes (*n* = 56), irrelevant study populations (*n* = 39), publication in languages other than English or Chinese (*n* = 5), or analyses limited to risk factors only (*n* = 20). A total of 12 studies were included, and one additional study was identified through manual searching, resulting in 13 studies included in the final meta-analysis. The study selection process is shown in the PRISMA flow diagram (Figure 1).

### 3.2 Characteristics of the included primary studies

Among the 13 studies included, all were conducted in China and published between 2019 and 2025. In terms of study design, nine studies were retrospective and four were prospective. All studies were carried out in large tertiary hospitals across different regions of China. The reported prevalence of severe *M. pneumoniae* pneumonia varied across studies, ranging from 18.8 to 91.6%. Detailed characteristics of the included studies are summarized in Table 1.

**Table 1:** Basic characteristics of the included literature.

Study	Year	Study design	Prevalence	Location of research	Predictors
He et al.	2025	Retrospective study	—	The First Hospital of Jilin University	ALT, age, AST, WBC, Lactate Dehydrogenase (LDH), length of stay
Gong et al.	2025	Retrospective study	22.31%	Kunming Children’s Hospital	Erythrocyte Sedimentation Rate (ESR), Platelet count (PLT), Interleukin 6 (IL6), and lung auscultation
Li et al.	2024	Retrospective study	25.9%	The Tianjin Children’s Hospital	Age, Decreased breath sounds, respiratory rate, duration of fever, length of stay, coinfection, ferritin, and LDH
Chang et al.	2022	Prospective study	36.1%	Wuxi Occupational Disease Prevention and Treatment Hospital	IgM, eosinophil proportion, eosinophil count, ESR and prealbumin
Liu et al.	2025	Retrospective study	91.6%	West China Second Hospital of Sichuan University	Duration of fever, wheezing, extrapulmonary complications, hemoglobin, pulmonary consolidation, mosaic sign, and bronchial occlusion
Xie et al.	2025	Prospective study	48.0%	The Wuhan Children’s Hospital	S100 A8/A9, C-Reactive Protein (CRP), PLT, APTT, the neutrophil-to-lymphocyte ratio (NLR), and CD4^+^CD25^+^ Treg
Zhang et al.	2024	Retrospective study	47.8%	Henan Children’s Hospital	Age, NLR, CRP, ESR, PLT, coinfection, pleural effusion, duration of fever and wheeze
Ye et al.	2025	Retrospective study	18.8%	Children’s Hospital of Soochow University	Duration of fever, D-dimer, PLT, CRP, LDH, and NLR
Kang et al.	2023	Retrospective study	65.1%	Shanxi Children′s Hospital	Duration of fever, PLT, LDH
Zhu	2019	Prospective study	24.8%	The First People’s Hospital of Changde	CRP, IgG, IgM, and LDH
Wu	2025	Retrospective study	32.6%	The Second Hospital of Shandong University	Duration of fever, CRP, LDH, ferritin, D-dimer and pleural effusion
Wang	2025	Prospective study	—	Nanshan District People’s Hospital of Shenzhen	SAT1, TNFRSF10B4, Nitrophenol, L-Arginine
Tang	2025	Retrospective study	—	Affiliated Hospital of Xinglin College, Nantong University	Duration of fever, CRP, LDH, AST and NLR

### 3.3 Predictors of the included models

Among the 13 included studies, more than 40 candidate predictors were identified. Fever duration, CRP, LDH, and NLR were the most frequently reported predictors and showed relatively consistent use across models. These variables may represent potential core predictors for future SMPP prediction model development. Table 2 summarizes predictors reported in at least two studies.

**Table 2:** Distribution of predictor variables in the model.

Predictor/study	Frequency (*n*)	He et al. (19)	Gong et al. (24)	Li et al. (44)	Chang et al. (45)	Liu et al. (25)	Xie et al. (20)	Zhang et al. (46)	Ye et al. (21)	Kang et al. (47)	Zhu (48)	Wu (49)	Wang (23)	Tang (22)
Age	3	★		★				★						
Duration of fever	7			★		★		★	★	★		★		★
Wheezing	2					★		★						
Length of stay	2	★		★										
Coinfection	2			★				★						
AST	2	★												★
PLT	5		★				★	★	★	★				
CRP	6						★	★	★		★	★		★
ESR	3		★		★			★						
Ferritin	2			★								★		
LDH	7	★		★					★	★	★	★		★
D-dimer	2								★			★		
NLR	4						★	★	★					★
IgM	2				★						★			
Pleural effusion	2							★				★		

### 3.4 Performance comparison of machine learning models

A total of 13 studies reporting diagnostic accuracy were included in the narrative synthesis. The sample sizes of the modeling datasets in the included studies ranged from 57 to 2,202. A variety of modeling approaches were applied, including LR, RF, SVM, and gradient boosting-based algorithms. The AUC of the optimal models reported in individual studies ranged from 0.777 to 0.975. One study performed external validation, whereas the remaining studies conducted internal validation using *k*-fold cross-validation or bootstrapping. Detailed information on model construction and validation is presented in Table 3.

**Table 3:** Model construction method and predictive performance.

Study	Modeling dataset	Validation dataset	Modeling methods	Validation method	Internal validation methods	Model performance	
						AUC	Calibration
He et al. (19)	2,202	643	LightGBM*, XGBoost, RF, KNN, LR	External verification	*K*-fold cross-validation	0.975 (0.964, 0.986)	Calibration curve
Gong et al. (24)	372	111	RF, LASSO, LR*	Internal verification	*K*-fold cross-validation	0.964 (0.945, 0.983)	Calibration curve
Li et al. (44)	932	400	LR*	Internal verification	Bootstrapping	0.862 (0.839, 0.886)	Hosmer–Lemeshow test
Chang et al. (45)	233	70	LR*	Internal verification	Bootstrapping	0.777 (0.699, 0.796)	Hosmer–Lemeshow test
Liu et al. (25)	562	168	LASSO, LR*	Internal verification	*K*-fold cross-validation	0.972 (0.936, 0.986)	Calibration curve
Xie et al. (20)	419	118	LightGBM*, Xgboost, LR, RF, KNN, SVM, DT, NB	Internal verification	*K*-fold cross-validation	0.953 (0.912, 0.976)	Calibration curve
Zhang et al. (46)	350	83	LR*	Internal verification	Bootstrap	0.870 (0.831, 0.888)	Calibration curve
Ye et al. (21)	387	96	LR, SVM, CDT, RF, GBDT, LightGBM, CatBoost*, XGBoost	Internal verification	*K*-fold cross-validation	0.934 (0.911, 0.951)	Calibration curve
Kang et al. (47)	307	92	LR*	Internal verification	Bootstrap	0.819 (0.798, 0.843)	Calibration curve
Zhu (48)	201	60	LR*	Internal verification	Bootstrap	0.850 (0.778, 0.923)	Calibration curve
Wu (49)	413	123	LR*	Internal verification	Bootstrap	0.952 (0.930, 0.975)	Calibration curve Hosmer–Lemeshow test
Wang (23)	57	—	RF*	—	—	0.920 (0.903, 0.947)	—
Tang (22)	190	127	GBM, LDA, NB, SVM, XGBoost*	Internal verification	*K*-fold cross-validation	0.920 (0.903, 0.947)	Calibration curve

CDT, classification decision tree; DT, decision tree; GBDT, gradient boosting decision tree; KNN, *K*-nearest neighbors; LightGBM, light gradient boosting machine; LR, logistic regression; LASSO, least absolute shrinkage and selection operator regression; LDA, linear discriminant analysis; NB, Naïve Bayes; RF, random forest; SVM, support vector machine; Xgboost, extreme gradient boosting; *, optimal model.

Most of the included studies compared the performance of multiple machine learning algorithms and identified the optimal model for predicting SMPP in children. He et al. (19) evaluated five algorithms, including LightGBM, XGBoost, RF, KNN, and LR, using the largest modeling dataset included in this review (*n* = 2,202). Among these approaches, the LightGBM model achieved the best predictive performance, with an AUC of 0.975. This study was also the only included study that performed external validation using an independent validation dataset. Xie et al. (20) compared multiple machine learning methods, including LightGBM, XGBoost, LR, RF, KNN, SVM, DT, and NB, and similarly found that the LightGBM model demonstrated the highest predictive performance, achieving an AUC of 0.953.

Several studies further evaluated ensemble learning approaches for SMPP prediction. Ye et al. (21) compared LR, SVM, CDT, RF, GBDT, LightGBM, CatBoost, and XGBoost models and reported that the CatBoost model achieved the highest predictive performance, with an AUC of 0.934. Tang (22) compared GBM, LDA, NB, SVM, and XGBoost models, among which the XGBoost model demonstrated the best performance, with an AUC of 0.920. Wang (23) established an RF-based prediction model and reported an AUC of 0.920 despite the relatively small sample size. Gong et al. (24) compared RF, LASSO regression, and LR models and found that the LR model achieved the best predictive performance, with an AUC of 0.964. Liu et al. (25) similarly combined LASSO regression with LR analysis and reported excellent discriminatory ability, with an AUC of 0.972.

Across studies, LR models was frequently used as a baseline modeling approach, with reported AUC values ranging from 0.777 to 0.952. In several cases, LR-based models showed comparable performance to machine learning approaches, particularly when feature selection or regularization methods were applied.

### 3.5 Meta-analysis of performance measures

**Figure 2:**
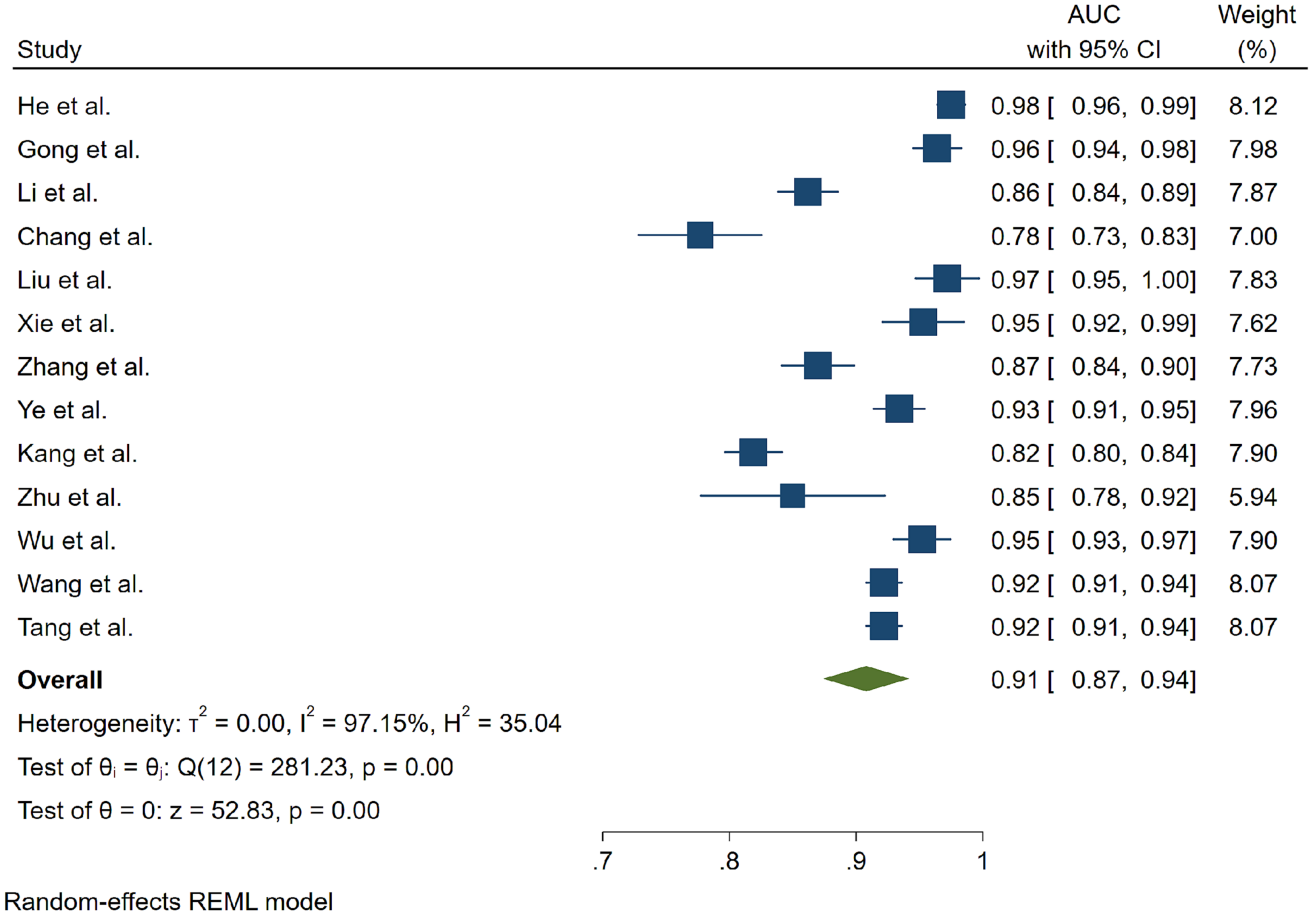
Forest plot of the area under the receiver operating characteristic curve for the risk prediction model.

**Figure 3:**
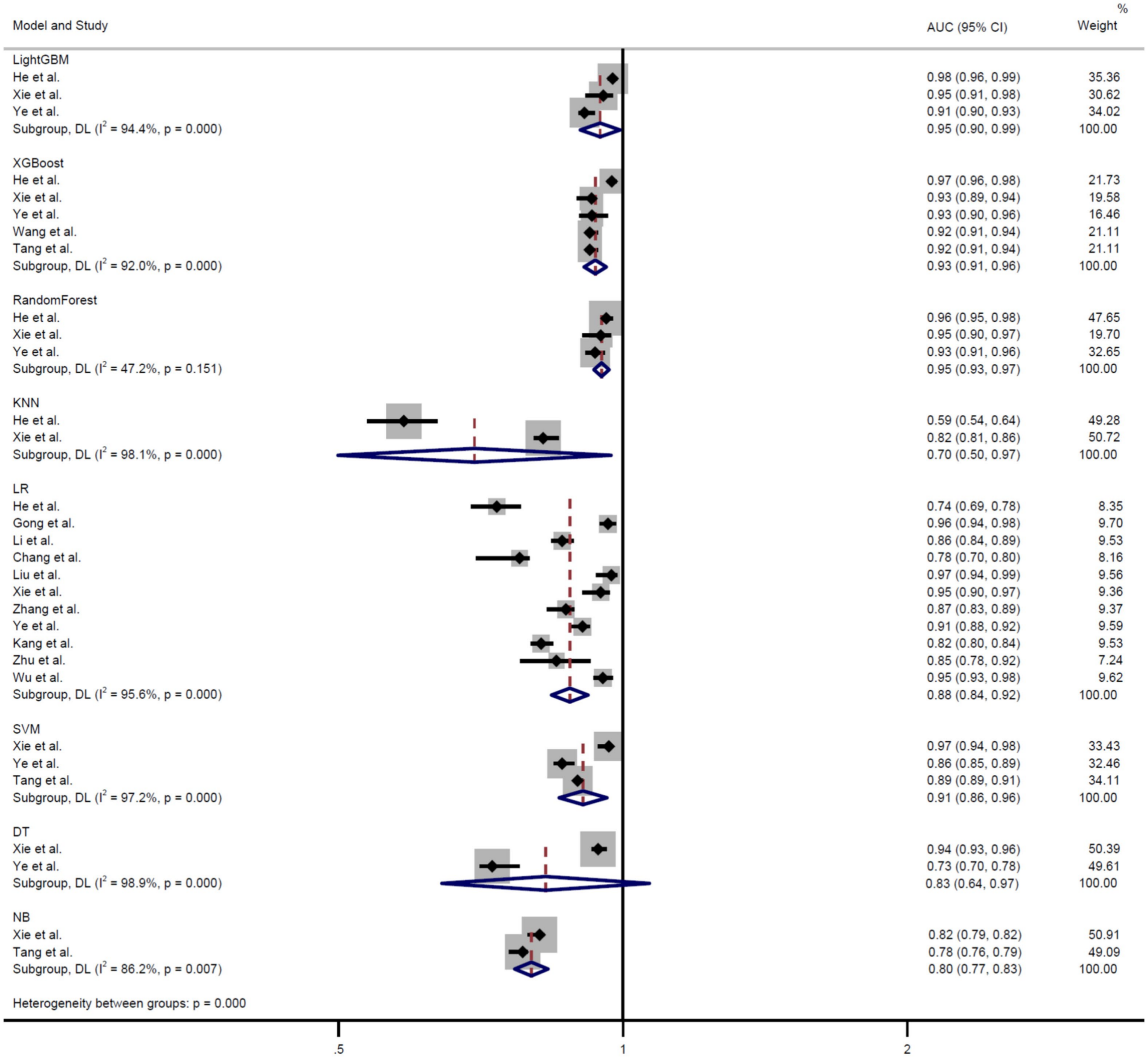
Forest plot of pooled AUCs for risk prediction models by modeling approach.

The forest plot summarizes the AUC values of the included studies. The pooled AUC was 0.91 (95% CI: 0.87–0.94) with a 95% prediction interval (PI) of 0.72–0.98 (Figure 2). Subgroup analyses stratified by modeling algorithms were conducted. The pooled AUCs were 0.95 (95% CI: 0.90–0.99; PI: 0.82–0.99; *I*^2^ = 98.8%, *p* < 0.001) for LightGBM, 0.94 (95% CI: 0.90–0.98; PI: 0.80–0.98; *I*^2^ = 95.7%, *p* < 0.001) for XGBoost, 0.95 (95% CI: 0.93–0.97; PI: 0.85–0.98; *I*^2^ = 47.2%, *p* = 0.151) for RF, 0.70 (95% CI: 0.50–0.97; PI: 0.45–0.88; *I*^2^ = 98.1%, *p* < 0.001) for KNN, 0.88 (95% CI: 0.84–0.92; PI: 0.70–0.95; *I*^2^ = 95.5%, *p* < 0.001) for LR, 0.91 (95% CI: 0.88–0.96; PI: 0.78–0.96; *I*^2^ = 97.2%, *p* < 0.001) for SVM, and 0.83 (95% CI: 0.64–0.97; PI: 0.60–0.92; *I*^2^ = 98.9%, *p* < 0.001) for DT (Figure 3). Among the evaluated approaches, ensemble learning algorithms such as XGBoost and Random Forest reported relatively high discriminatory performance across studies. However, substantial heterogeneity was observed across studies; therefore, the pooled estimate should be interpreted with caution.

### 3.6 Sensitivity analysis

**Figure 4:**
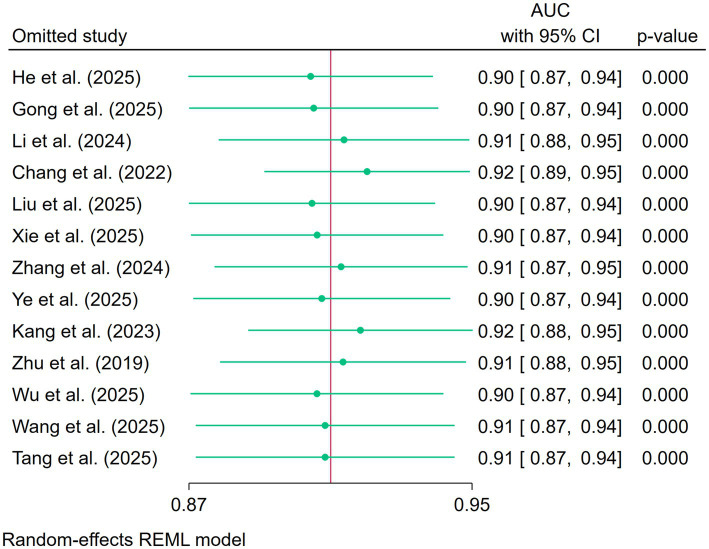
Leave-one-out sensitivity analysis of pooled AUC estimates.

Sensitivity analysis was conducted using a leave-one-out approach under the random-effects REML model. The pooled AUC estimates ranged from 0.90 to 0.92 after sequential omission of individual studies. All recalculated pooled estimates remained statistically significant (all *p* < 0.001). Detailed results of the sensitivity analysis are presented in Figure 4.

### 3.7 Quality assessment of included studies

In general, most included studies had a high or unclear risk of bias, with only one study rated as low risk according to the PROBAST assessment (Table 4). Across domains, high or unclear risk of bias was most frequently observed in the analysis domain, while the domains of participants, predictors, and outcomes showed a mixture of low, high, and unclear risk. Low to high concerns related to applicability were identified across studies, although most studies were judged to have low concern.

**Table 4:** Risk of bias assessment.

Study	Risk of bias				Applicability			Overall	
	Participants	Predictors	Outcome	Analysis	Participants	Predictors	Outcome	ROB	Applicability
He et al. (19)	+	+	+	+	+	+	+	+	+
Gong et al. (24)	−	+	?	−	+	+	?	−	−
Li et al. (44)	+	+	?	?	+	+	?	?	+
Chang et al. (45)	−	+	?	−	+	+	?	−	+
Liu et al. (25)	−	+	?	?	+	+	?	?	+
Xie et al. (20)	+	+	+	−	+	+	+	−	+
Zhang et al. (46)	−	+	−	−	+	+	+	−	+
Ye et al. (21)	+	+	+	?	+	+	+	?	+
Kang et al. (47)	−	+	?	−	+	+	−	−	+
Zhu (48)	+	+	?	?	+	+	−	?	+
Wu (49)	−	−	?	−	+	+	+	−	+
Wang (23)	+	−	−	−	+	−	−	−	?
Tang (22)	+	+	−	−	+	+	−	−	+

ROB, risk of bias; “+” = low ROB/low concern regarding applicability; “−” = high ROB/high concern regarding applicability.

“?” = unclear ROB/unclear concern regarding applicability.

Potential small-study effects were assessed using Egger’s regression test under a random-effects REML model. The results suggested the presence of small-study effects (*p* = 0.006). Therefore, a trim-and-fill analysis was further performed. The trim-and-fill method imputed four potentially missing studies, and the pooled effect estimate remained statistically significant after adjustment, although the corrected effect size was attenuated compared with the observed estimate.

## 4 Discussion

### 4.1 Common predictors for progression to SMPP

This study systematically reviewed ML-based prediction models for SMPP in children and summarized the reported model performance across studies. By summarizing predictors incorporated across the included models, we aimed to identify commonly used variables and provide evidence to support early risk stratification in pediatric clinical practice. Although modeling strategies varied across studies, substantial overlap was observed in the predictors selected.

Across the 13 included studies, more than 40 candidate predictors were identified, among which fever duration, CRP, LDH, and NLR were repeatedly incorporated into different prediction models, suggesting a relatively consistent consensus regarding their relevance in predicting the risk of SMPP in children. Fever duration, included in seven studies, was one of the most frequently used clinical predictors. Prolonged fever may reflect insufficient immune clearance of *M. pneumoniae*, indicating sustained inflammatory activity and delayed disease control, which in turn may contribute to aggravated pulmonary inflammation and tissue injury characteristic of SMPP (26). CRP was incorporated as a predictor in six studies. As a widely used acute-phase inflammatory marker, elevated CRP levels reflect an intensified systemic inflammatory response, and previous studies (27–30) have demonstrated a close association between increased CRP concentrations and radiological progression, complication development, and poor therapeutic response in pediatric MPP, supporting the role of excessive inflammation in the progression to severe disease. LDH was included in seven studies and represented one of the most commonly used laboratory predictors. Yanhong et al. (31) explored the predictive value of LDH in children with MPP. Results from restricted cubic spline analysis indicated a nonlinear dose–response relationship between continuously elevated LDH levels and the incidence of SMPP. Moreover, decision curve analysis demonstrated the significant clinical utility of LDH in predicting severe MPP. Elevated LDH levels are thought to indicate cellular injury and pulmonary tissue damage resulting from severe inflammatory processes, and their consistent inclusion across models underscores the potential value of LDH as a surrogate marker of disease severity and extent of lung involvement in SMPP (32). NLR was incorporated as a predictive variable in four studies. By integrating information from neutrophil-mediated innate immunity and lymphocyte-mediated adaptive immunity, NLR serves as a convenient indicator of immune imbalance. Evidence from previous research suggests (33, 34) that elevated NLR is associated with enhanced inflammatory responses and disease progression in pediatric MPP, implying that dysregulated immune responses may play a role in the exacerbation of SMPP.

In addition to these frequently reported predictors, some models included imaging features such as pulmonary consolidation, pleural effusion, or bronchial occlusion, as well as extrapulmonary complications. However, these predictors were less consistently reported and may be influenced by differences in diagnostic resources and institutional practices. Overall, the convergence of predictors across studies indicates that SMPP prediction models predominantly rely on routinely available clinical indicators and laboratory inflammatory markers. This consistency supports the feasibility of applying such models in routine pediatric practice, while also highlighting the need for standardized predictor selection and reporting in future model development.

### 4.2 Predictive accuracy and methodological considerations

In the included studies, model performance was predominantly evaluated using the AUC, which is the most commonly reported indicator of discrimination in prediction model research (35). In general, higher AUC values indicate better discriminatory ability. The pooled results of the present meta-analysis showed a combined AUC of 0.91 (95% CI: 0.87–0.94). However, substantial heterogeneity was observed across studies, including differences in study populations, predictor selection, outcome definitions, modeling strategies, and validation methods. Therefore, the pooled AUC should be interpreted as an exploratory summary of reported model discrimination rather than a definitive estimate of predictive performance in clinical practice. Subgroup analyses stratified by modeling algorithms further revealed differences in predictive performance across model types. Among the evaluated approaches, XGBoost and Random Forest demonstrated the best predictive performance, with pooled AUCs of 0.94 (95% CI: 0.90–0.98) and 0.95 (95% CI: 0.93–0.97), respectively. These findings suggest that ensemble learning methods may be more effective in capturing complex nonlinear relationships and interactions among predictors in SMPP risk stratification. In contrast, KNN models showed relatively limited discriminatory ability, while logistic regression and SVM models demonstrated moderate to good performance. Despite the favorable discrimination observed, substantial heterogeneity was noted across most model subgroups, as reflected by high *I*^2^ values. This heterogeneity is not unexpected in prediction model meta-analyses and may be attributed to variations in study populations, predictor definitions, outcome criteria, modeling strategies, and validation methods. Differences in disease severity thresholds, laboratory cut-off values, and institutional diagnostic practices may also contribute to between-study variability. Therefore, although pooled AUC estimates indicate good overall performance, caution is warranted when interpreting and applying these results in different clinical contexts.

Model validation is a critical component of prediction model development. However, in the included studies, most models relied primarily on internal validation methods, while only one study performed external validation. It is important to distinguish between model development performance, internal validation performance, and external validation performance. Performance estimates derived from development datasets or internal validation procedures (e.g., *k*-fold cross-validation or bootstrapping) are generally more optimistic than those obtained from independent external datasets. Therefore, the pooled AUC estimates in the present study may overestimate the predictive performance of these models in real-world clinical settings.

In addition, many of the included models were developed using retrospective single-center data, which may further limit generalizability across different clinical settings and pediatric populations. External validation using independent and preferably multicenter datasets remains essential for evaluating model robustness and transportability, particularly for complex machine learning algorithms. To further examine the stability of the pooled results, leave-one-out sensitivity analyses were conducted. The pooled AUC estimates remained within a narrow range after sequential omission of individual studies, indicating that the overall results were not substantially influenced by any single study.

### 4.3 Implications for future research

With the continued advancement of machine learning in predictive medical modeling, its application in assessing the risk of SMPP in children has garnered increasing interest. This study systematically reviews SMPP risk prediction models developed using machine learning algorithms. Results indicate that existing models generally exhibit consistently high discriminatory performance, demonstrating the ability to effectively integrate clinical manifestations, laboratory parameters, and imaging features to quantify the risk of disease progression. Recent studies have also highlighted the potential value of non-invasive imaging techniques, including quantitative CT analysis and radiomics-based approaches, in the diagnosis and prognostic assessment of *M. pneumoniae* pneumonia (36, 37). The integration of imaging-derived features with machine learning models may further improve risk stratification and early identification of severe cases. However, differences remain across studies in predictor selection, modeling algorithms, and internal validation strategies, which may affect model performance. Methodological research has emphasized (38) that machine learning models are not inherently superior to traditional approaches; rather, their performance advantages depend on rigorous modeling procedures and transparent reporting. Therefore, future studies should continue to adopt machine learning–based frameworks while adhering more closely to established guidelines such as TRIPOD and PROBAST. Detailed reporting of predictor selection rationale, model training procedures, and internal and external validation methods is essential to enhance reproducibility and credibility.

Moreover, several models included in this review utilized ensemble learning algorithms, reflecting the strength of these methods in capturing nonlinear relationships and complex interactions among variables. Tomita et al. (39) have demonstrated that XGBoost and random forest models show favorable generalizability in disease risk prediction, providing both theoretical and practical support for their broader application in SMPP risk assessment. In future research, a more rigorous indicator selection process can be adopted. We can evaluate the stability and reliability of the importance of each predictors, and combine cross-validation to select the final indicators for inclusion in the model. This approach will help build more robust clinical prediction tools that can be effectively applied to new patients. Interpretability remains a critical factor for the clinical translation of machine learning-based prediction tools (40).

Techniques such as SHAP values and feature contribution rankings can help clarify the clinical relevance of key predictors, thereby increasing clinicians’ understanding of and confidence in model outputs (41). For complex machine learning models such as XGBoost and Random Forest, which are often considered “black-box” methods, interpretability should be regarded as a necessary component for clinical use rather than an optional feature. This is especially important in pediatric clinical decision-making, where model transparency is closely linked to clinician trust and practical applicability (42). From a clinical implementation perspective, reporting model performance metrics alone is insufficient to support real-world adoption. Greater attention should be directed toward translating machine learning prediction models into actionable clinical decision-support tools. Thus, future SMPP prediction model research should place stronger emphasis on the clinical presentation and usability of model outputs, allowing clinicians to interpret and apply predictions intuitively.

Additionally, ongoing model updating and validation using multicenter real-world data may improve stability and applicability across diverse regions and pediatric populations, thereby providing more reliable evidence to support early identification and clinical management of children with SMPP. Future studies should provide explicit justification of sample size and events-per-variable (EPV) considerations during model development, as insufficient sample size relative to model complexity remains a key source of instability and potential overfitting in prediction models, particularly in machine learning frameworks. Addressing the lack of external validation should be a priority for future research. Multicenter studies are needed to improve the applicability of prediction models. The development of open-access validation datasets would further facilitate independent evaluation and transparent comparison of model performance across different settings. Notably, compared with well-established international databases such as the National Health and Nutrition Examination Survey (NHANES) (43), there is a lack of large-scale and publicly accessible clinical databases for pediatric SMPP in China, which may partly explain the limited external validation in existing studies.

### 4.4 Limitations

This study has several limitations. First, the literature search was restricted to studies published in English and Chinese, which may have led to the omission of relevant studies in other languages. Second, substantial heterogeneity was observed across included studies in terms of study design, populations, predictor selection, and modeling approaches, which is inherent in prediction model research. Third, most models were developed using retrospective data with limited external validation, which may limit their applicability across different clinical settings. In addition, all included studies were conducted in China, which may further limit the generalizability of these findings to other regions. Finally, although Egger’s test suggested potential small-study effects, the interpretation of publication bias in prediction model meta-analyses remains challenging, and these findings may also be influenced by methodological heterogeneity and optimistic internal validation.

## 5 Conclusion

Through a systematic search and selection process, this study included 13 prediction models for SMPP in children and summarized their predictors, modeling methods, and reported performance. The pooled AUC was 0.91, indicating generally good discriminatory ability across included models. Subgroup analyses showed that models based on XGBoost and Random Forest tended to report higher AUC values compared with other approaches. However, heterogeneity across studies and limited external validation were observed. These factors should be considered when interpreting the pooled results and applying the findings in clinical settings. Further studies with multicenter data and external validation are needed to support more robust model evaluation.

## Data Availability

The original contributions presented in the study are included in the article/Supplementary material, further inquiries can be directed to the corresponding author.
