# Supplementary material for: Machine learning-based prediction models for severe Mycoplasma pneumoniae pneumonia in Chinese children: a systematic review and meta-analysis of prediction model performance
Source: Front Public Health. 2026 Jun 3;14:1793116. doi: 10.3389/fpubh.2026.1793116 (PMC13272165; doi:10.3389/fpubh.2026.1793116)
Supplement: Supplementary file 1 [file Table_1.DOCX]

| **Table S1.** Search Strategy | |
| --- | --- |
| Database | Search Strategy |
| PubMed | ① ("Mycoplasma pneumoniae pneumonia" OR "mycoplasma pneumonia" OR "severe Mycoplasma pneumoniae pneumonia" OR "SMPP") |
|  | ②("machine learning" OR "artificial intelligence" OR "deep learning" OR "random forest" OR "decision tree" OR "XGBoost"OR "LightGBM"OR "Logistic Regression"OR "Naïve Bayes"OR "Artificial Neural Network"OR "Convolutional Neural Network" OR "Support Vector Machine"OR "Nomogram" OR "prediction model" OR "risk prediction" OR "risk factor") |
|  | ③#1 AND #2 |
| Web of Science | ① TS=("Mycoplasma pneumoniae pneumonia" OR "mycoplasma pneumonia" OR "severe Mycoplasma pneumoniae pneumonia" OR SMPP) |
|  | ②TS=("machine learning" OR "artificial intelligence" OR "deep learning" OR "random forest" OR "decision tree" OR XGBoost OR LightGBM OR "logistic regression" OR "naive bayes" OR "artificial neural network" OR "convolutional neural network" OR "support vector machine" OR nomogram OR "prediction model" OR "risk prediction" OR "risk factor*") |
|  | ③#1 AND #2 |
| Embase | ①('mycoplasma pneumoniae pneumonia'/exp OR 'mycoplasma pneumonia' OR 'mycoplasma pneumoniae pneumonia' OR 'severe mycoplasma pneumoniae pneumonia' OR smpp):ti,ab,kw |
|  | #2('machine learning'/exp OR 'artificial intelligence'/exp OR 'deep learning'/exp OR 'random forest' OR 'decision tree' OR xgboost OR lightgbm OR 'logistic regression' OR 'naive bayes' OR 'artificial neural network'/exp OR 'convolutional neural network'/exp OR 'support vector machine' OR nomogram OR 'prediction model' OR 'risk prediction' OR 'risk factor*'):ti,ab,kw |
|  | ③#1 AND #2 |
| CINAHL | ① ("Mycoplasma pneumoniae pneumonia" OR "mycoplasma pneumonia" OR "severe Mycoplasma pneumoniae pneumonia" OR "SMPP") |
|  | ②("machine learning" OR "artificial intelligence" OR "deep learning" OR "random forest" OR "decision tree" OR "XGBoost"OR "LightGBM"OR "Logistic Regression"OR "Naïve Bayes"OR "Artificial Neural Network"OR "Convolutional Neural Network" OR "Support Vector Machine"OR "Nomogram" OR "prediction model" OR "risk prediction" OR "risk factor") |
|  | ③#1 AND #2 |
| CNKI | ① SU=("肺炎支原体肺炎" OR "支原体肺炎"OR "重症肺炎支原体肺炎" OR "重症支原体肺炎") |
|  | ② SU=("机器学习" OR "人工智能" OR "深度学习" OR "随机森林" OR "决策树" OR "支持向量机" OR "逻辑回归" OR "朴素贝叶斯" OR "人工神经网络" OR "卷积神经网络" OR "预测模型" OR "风险预测" OR "危险因素" OR "列线图") |
|  | ③#1 AND #2 |
| Wanfang Database | ①主题=("肺炎支原体肺炎" OR "支原体肺炎"OR "重症肺炎支原体肺炎" OR "重症支原体肺炎") |
|  | ②主题=("机器学习" OR "人工智能" OR "深度学习" OR "随机森林" OR "决策树" OR "支持向量机" OR "逻辑回归" OR "朴素贝叶斯" OR "人工神经网络" OR "卷积神经网络" OR "预测模型" OR "风险预测" OR "危险因素" OR "列线图") |
|  | ③#1 AND #2 |
| VIP Database | ①Topic=("肺炎支原体肺炎" OR "支原体肺炎"OR "重症肺炎支原体肺炎" OR "重症支原体肺炎") |
|  | ②Topic=("机器学习" OR "人工智能" OR "深度学习" OR "随机森林" OR "决策树" OR "支持向量机" OR "逻辑回归" OR "朴素贝叶斯" OR "人工神经网络" OR "卷积神经网络" OR "预测模型" OR "风险预测" OR "危险因素" OR "列线图") |
|  | ③#1 AND #2 |
